# MicroRNA-448 inhibits the progression of retinoblastoma by directly targeting ROCK1 and regulating PI3K/AKT signalling pathway

**DOI:** 10.3892/or.2022.8351

**Published:** 2022-06-20

**Authors:** Shen Wu, Nanping Ai, Qian Liu, Jingxue Zhang

Oncol Rep 39: 2402–2412, 2018; DOI: 10.3892/or.2018.6302

Following the publication of this paper, it was drawn to the Editors' attention by a concerned reader that three data panels featured in the flow cytometric plots shown in Figs. 5D and 6D, and several panels from the cell invasion assays shown in Figs. 5C and 6C, were strikingly similar to data appearing in different form in other articles by different authors. Owing to the fact that the contentious data in the above article were already under consideration for publication prior to its submission to *Oncology Reports*, the Editor has decided that this paper should be retracted from the Journal. After having been in contact with the authors, they agreed with the decision to retract the paper. The Editor apologizes to the readership for any inconvenience caused.

